# Study of Pd-based catalysts within red algae-derived polysaccharide supports in a Suzuki cross-coupling reaction

**DOI:** 10.1039/c8ra08408d

**Published:** 2018-11-12

**Authors:** Adi Wolfson, Shira Biton, Oshrat Levy-Ontman

**Affiliations:** Green Process Center, Sami Shamoon College of Engineering Bialik 56 Beer-Sheva Israel adiw@sce.ac.il oshrale@sce.ac.il

## Abstract

Simple palladium complexes were heterogenized into red algae derived polysaccharide supports, and the effects of polysaccharide, catalyst and solvent types on the performances in a Suzuki cross-coupling reaction were tested. It was found that using palladium salts with sodium triphenylphosphine trisulfonate (TPPTS) as a ligand supported on ι-carrageenans and ethanol as the solvent yielded the best systems. Moreover, the conversion rates of these heterogeneous systems were higher than their homogeneous analogues, and they were easily recycled five times. SEM-EDS analysis of Pd(OAc)_2_(TPPTS)_2_ that was immobilized on ι-carrageenan support was also performed, demonstrating that the system has a porous structure composed of Pd complex that was embedded within the ι-carrageenan. In addition, both ι-Pd(OAc)_2_(TPPTS)_2_ and ι-Pd(OAc)_2_ systems, were composed of nanoparticles, as proven by TEM analysis.

## Abstract 2

Simple palladium complexes were heterogenized into red algae derived polysaccharide supports, and the effects of polysaccharide, catalyst and solvent types on the performances in a Suzuki cross-coupling reaction were tested.

## Graphical abstract

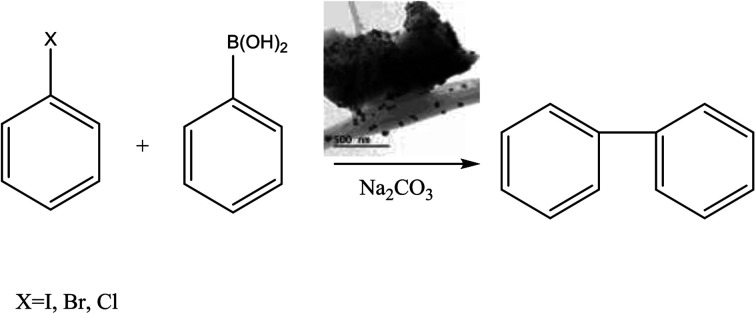

## Introduction

Homogeneous transition metal complexes (TMCs) are very attractive catalysts for many organic transformations.^1^ They are usually designed and tailored to be very specific, active and selective, compared to their analogous heterogeneous catalysts. Yet, regardless of their advantages, the tedious separation of these catalysts from the product at the end of the reaction and the difficulty to recycle them, have led to the development of different approaches to heterogenize homogeneous TMCs, thus combining the advantages of homogeneous and heterogeneous catalysis.^2–4^

TMC heterogenization can be performed *via*, among others, chemical immobilization, physical occlusion or entrapment, within organic and inorganic supports. Besides the demand to avoid the TMC leaching from the support, there are several other considerations that should be taken into account while choosing a heterogenization route, such as the cost and the ease of the heterogenization procedure, the extent to which the heterogeneous catalyst preserves the performance of the homogenous mother complex, and mass transfer limitations through the support. In this regard, complicated modification of the complex before its immobilization and loss of performance due to chemical modification, are the two main disadvantages of heterogenization *via* TMC bonding to a support.^2–4^ On the other hand, occlusion or entrapment of the complex in a solid matrix without any modification, usually requires to form a relatively dense matrix structure in order to ensure that the complex will not leach out, thus often resulting in mass transfer limitations of both the reactants and the products. Finally, supporting the TMC on the external and/or the internal surface of the solid matrix, requires different manipulations to prevent the leaching of the complex, such as using a solvent that does not dissolve the complex. At last, although catalysis is a fundamental pillar of green chemistry, environmental concerns and restrictions also oblige to check the impacts of catalyst preparation, separation and recycling on the natural environment.^5,6^ Therefore, the search for an ultimate heterogeneous catalyst, composed of an environmental friendly support and which can be used in an environmentally benign fashion, is of high concern.

Biopolymers are biodegradable organic polymers that are derived from renewable sources such as bacteria, plants and algae. These bioactive natural products have applications in various industries, such as pharmaceutical, biomedical, cosmetics and food, and also as fibers and building blocks for many materials.^7,8^ An emerging class of biopolymers that has attracted much attention, are polysaccharides produced by red algae. One of the most studied family among this class are those derived from red seaweed, designated as carrageenans, and the most widely known carrageenans in the industry are iota (ι), kappa (κ) and lambda (λ) forms.^9,10^ Most of the spread commercially renewable biopolymers are soluble in water and have negligible solubility in organic solvents, even in highly polar and protic solvents such as methanol and ethanol.^11^ In addition, they can form hydrogels in water in the presence of different agents (*e.g.*, metals or bases). Thus, they seem to be also very attractive candidates to serve as polymeric supports for catalysis.

Indeed, polysaccharides have been used as catalysts by themselves and as supports for metal catalysts. For example, carrageenans, that bear both hydroxyl and anionic sulfate groups, were used as green heterogeneous Lewis acid catalysts in water and different organic solvents^12,13^ and chitosan that bears amine groups structure was used as a heterogeneous catalyst in Michael additions.^14^ In addition, zinc cations supported on carrageenan magnetic nanoparticles were used as a green and efficient catalytic system for a one-pot three-component synthesis of quinoline derivatives.^13^ Furthermore, various heterogeneous palladium catalysts were prepared with a variety of polysaccharides as supports using different techniques, and their performances were tested in different reactions. For instance, polysaccharide aerogel microspheres were used to support thin water layer with water soluble palladium catalyst (*i.e.*, supported aqueous phase catalysis),^15^ and the heterogeneous catalyst was employed in the substitution of an allyl carbonate with morpholine.^16,17^ In addition, chitosan-supported palladium nanoparticles were tested as a catalyst in nitrophenol degradation,^18^ while nanoparticles of palladium supported on cellulose^19^ or on agar/pectin^20^ or agar^21^ were tested in catalytic hydrogenations. Finally, various palladium complexes and nanoparticles supported on polysaccharide matrices were also synthesized and used in C–C coupling reactions,^22^ such as Heck^23–27^ and Suzuki reactions.^20,21,25,28–36^ Yet, most of these palladium-based heterogeneous catalysts were prepared through multistep and tedious procedures.

The synthesis of biaryles *via* Suzuki cross-coupling with Pd-based polysaccharides systems, either Pd(0) or Pd(ii), has been the focus of many research groups. Primo *et al.* reported the synthesis of palladium nanoparticles in the matrix of alginate, by chelating of Na_2_PdCl_4_ to the carboxylate groups of alginate, which was followed by reduction of the palladium cation during dehydration of the Pd-alginate composite.^28^ The conversions of the catalyst in Suzuki reaction in DMF were relatively high (up to 98%), and the heterogeneous catalyst was successfully recycled with some loss of activity. In another research, Chen *et al.* reported a system of terpyridine–palladium(ii) complex that was attached to xylan-type hemicelluloses and reduced to nanoparticles.^29^ Again, the catalyst yields were high (>90%), using alcohols as solvent and activated halobenzanes, and was recycled for six times with minor loss of activity. Makhubela *et al.* synthesized chitosan and chitosan-based ligands that were used to immobilize PdCl_2_(COD) to form chitosan-supported Pd(ii) catalysts.^30^ The Suzuki reactions with these catalysts were performed in xylene at a relatively high temperature, yielding mild conversions. Chitosan was also employed to support palladium nanoparticles by Cotugno *et al.*, using electro-synthesis, and the catalyst that was tested in ionic liquids as solvent yielded high conversions just with the addition of water to the reaction mixture.^31^ In another work, Yi *et al.* reported on the preparation of a bead-type chitosan-supported ligand-free Pd(0) catalyst using a simple reduction process, and the prepared catalyst was studied in the Suzuki cross-coupling reaction in water, using microwave irradiation.^32^ As expected, the use of microwave irradiation accelerated the reaction, reaching 4–120 fold tern-over-frequencies (TOF) compared to the previous tested catalysts.^28–32^ In addition, the new system was also successfully recycled for 5 times without any loss of activity. Finally, recently, Baran *et al.*, reported on several methods to prepare palladium-based polysaccharide catalysts that were tested in a Suzuki reaction under microwave irradiation and without solvent, as very environmentally friendly systems.^20,21,33–39^ The first system was prepared by mixing Na_2_PdCl_4_ with chitosan–*Ulva* composite beads,^33^ whereas in the second type of system, amino functionalized polysaccharides: starch,^34^ agar^21^ and cellulose,^35^ were prepared by interaction of the hydroxyl groups on the polysaccharide with 3-aminopropyl triethoxysilane, which was followed by formation of Schiff base that was coordinated to Na_2_PdCl_4_. In a third system, chitosan/cellulose composite was mixed with Na_2_PdCl_4_, followed by reduction of the metal with NaBH_4_,^36^ while a cellulose/agar composite that was mixed with PdCl_2_ and reduced under reflux without any reduction agent using ultrasound in water,^37^ yielded palladium nanoparticles. All of these new heterogeneous catalysts were very active, yielding 5 times higher TOF compare to the reaction in water under microwave irradiation^21^ and up to 500 times TOF compare to the reactions under conventional heating.^29^

**Fig. 1:**
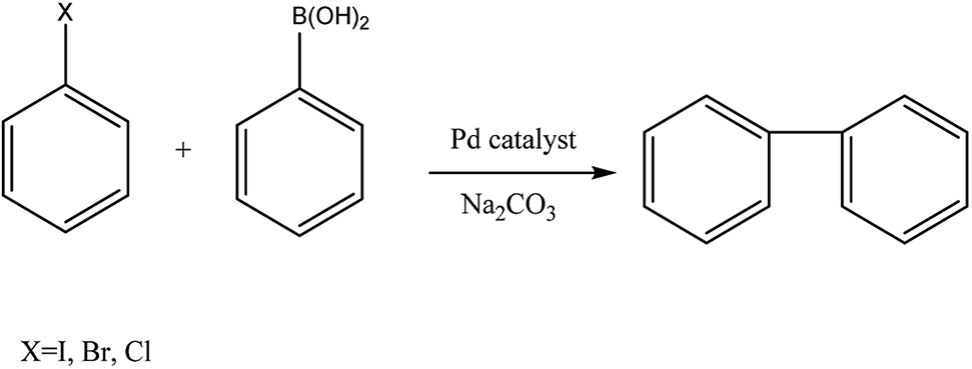
Suzuki cross-coupling of halobenzene and phenylboronic acid.

We recently reported on simple procedure to immobilize the complex PdCl_2_(TPPTS)_2_ (TPPTS = sodium triphenylphosphine trisulfonate) in various red-algae polysaccharides, where the heterogeneous system was efficiently used in Suzuki cross-coupling of halobenzens and phenylboronic acid (Fig. 1) in ethanol, yielding high conversions but mild TOFs. Moreover, it was demonstrated that the complex was attached to the polysaccharide by new bond between the sulfonate groups of TPPTS and the hydroxyl group of the polysaccharide, thus preventing the complex to leach to the solvent and allowing catalyst recycling.^38^

Herein, we report on the use of this new palladium–polysaccharide system as heterogeneous catalyst in the Suzuki cross-coupling of halobenzenes with phenylboronic acid (Fig. 1). Various palladium salts and complexes were tested, together with sodium carbonate as co-catalyst, in a variety of solvents. The effects of solvent type, polysaccharide type and palladium catalyst type on both the leaching of the homogeneous catalyst and the reaction conversion were studied, together with the catalyst separation and recycling process. In addition, preliminary structural characterization of Pd(OAc)_2_ and Pd(OAc)_2_(TPPTS)_2_ supported on ι carrageenan were also investigated.

## Experimental

### Reagents

All polysaccharides and other chemicals (analytical grades) were purchased from Aldrich. The polysaccharide derived from *Porphyridium* sp. (P) was given as a gift for research purposes by Frutarom Ltd, Israel.

### Catalyst preparation

In a typical procedure, 10 μmol of palladium salt (PdCl_2_ or Pd(OAc)_2_) were added to a glass vial together with 3 mL solution that contained 30 μmol of the ligand (TPP or TPPTS), and mixed at room temperature for 5 min. When TPPTS was used it was dissolved in DDW while TPP was dissolved in an ethanol : DDW solution (1 : 4 volume ratio). Then, the solution with the complex was added to a 15 mL polypropylene tube together with 3 mL of 1% wt polysaccharide solution in distilled water, sealed and mixed by vortexing for homogenization. In the next step, the tube was frozen at −20 °C for 24 h, until all the liquid froze. Then, the sealing was removed, and the tube was covered with paraffin sheet that was pierced with a disposable toothpick. The tube was placed in a lyophilizer for 48 h. At the end of the process the dried “sponge like” catalyst was cut into ∼1 cm × 1 cm square pieces and added to the reaction mixture.

### Reaction procedure

In a typical procedure, 10 μmol of palladium catalyst (homogeneous or heterogeneous) were added to a glass vial with 5 mL solvent together with 0.5 mmol halobenzene, 0.75 mmol phenylboronic acid and 0.6 mmol Na_2_CO_3_. The mixture was placed in a preheated oil bath at 60 °C and magnetically stirred for 24 h. At the end of the reaction, the reaction mixture was cooled and analyzed by GC using a HP-5 column to determine conversion.

### Leaching tests

Leaching of the catalysts was tested in three ways: (1) performing a second reaction after the removal of the catalyst from the original reaction mixture and the addition of the catalyst to a fresh reaction mixture with the corresponding amounts of fresh substrates and sodium carbonate, then checking whether the catalytic performance was comparable; (2) proceeding with the reaction after the removal of the catalyst by running the reaction mixture under similar conditions for an additional 24 h, to check whether the conversion increases with time; and (3) by doing an inductively-coupled plasma optical emission spectrometry (ICP-OES) (Arcos, Spectro) analysis of the reaction medium after a 24 h reaction time and the removal of the catalyst, to check for palladium leftovers in the solution.

### Catalyst recycling

Catalyst recycling was performed by the addition of the recovered catalyst to a solution with similar amounts of fresh substrates and base and running the reaction mixture under similar reaction conditions for an additional 24 h.

### Scanning electron microscope (SEM) and energy dispersive X-ray spectrometry (EDS)

Scanning electronic microscopy (SEM) and elemental analysis of ι-Pd(OAc)_2_(TPPTS)_2_ were performed with lyophilized samples that were previously coated with gold, using FEI Verios 460L XHR (extreme high resolution, Hillsboro, OR, USA) scanning electron microscope, equipped with energy-dispersive X-ray spectroscopy (EDS, Oxford Instruments). The acceleration voltage was 20 kW.

### Transmission electron microscope (TEM) analysis

High-resolution transmission electron microscopic (HRTEM) micrographs of ι-Pd(OAc)_2_(TPPTS)_2_ system were obtained using an EFI Talos F200C transmission electron microscope (TEM) operated at 200 kV at room temperature. The HRTEM micrograph for the ι-Pd(OAc)_2_ preparation was obtained by Analytical TEM JEOL JEM 2100F and the element determination was obtained by JEOL 50 mm^2^ Si(Li) detector, solid angle 0.24 rad, energy resolution of 133 eV (Mn K edge). The samples were prepared by deposition of a drop of ethanol suspension of the crushed solid catalyst on a carbon-coated Cu grid.

## Results and discussion

Suzuki cross-coupling of aryl halides and aryl boronic acids has become of great interest as a powerful tool in the synthesis of biaryls, useful building blocks in a range of pharmaceuticals, herbicides and polymers.^39–42^ Various palladium catalysts were reported to be suitable for this synthesis, the majority of reports involving soluble palladium salts and complexes and their heterogeneous analogues.^43–45^ As previously stated, the reaction medium, which plays a critical role in most organic reactions, also often controls the leaching of the catalyst from the support. Yet, the choice of the solvent in Suzuki reactions is even more crucial, since it has to dissolve together polar and apolar organic substrates, an inorganic base and a metal catalyst. Thus, the low solubilities of arylhalides in water as well as the low solubility of phenylbronic acid and inorganic base in apolar organic solvents, have led to the use of hazardous polar organic solvents such as DMF and DMSO as reaction media.^46^

The investigation initiated with the homogenous cross-coupling of iodobenzene and phenylboronic acid (Fig. 1) with several simple and commercial available palladium catalysts in ethanol (Table 1, entries 1–4). As biphenyl can be also synthesized *via* homo-coupling of phenylboronic acid or halobenzene, (*i.e.* Ullmann coupling), a reference reaction without the addition of catalyst was first tested and did not yield any biphenyl. In addition, although the reaction with simple palladium salts, such as palladium chloride and palladium acetate (entries 1 and 2, respectively), yielded high performance, it is well known that their use is also accompanied with deactivation of the catalysts due to formation of palladium black.^47^ Addition of phosphine ligands, such as triphenyl phosphine (TPP), can stabilize the catalyst and prevent its deactivation.^48^ Moreover, as our intention was to heterogenize the complex, a homogeneous reaction with the water soluble phosphine TPPTS,^49^ was also tested, since it was expected that using this ligand will lead to lower leaching to organic solvents. Hence, four types of complexes, PdCl_2_(TPP)_2_, Pd(OAc)_2_(TPP)_2_, PdCl_2_(TPPTS)_2_ and Pd(OAc)_2_(TPPTS)_2_, were tested in a homogeneous reaction in ethanol (entries 3–6, respectively), where both complexes with the ligand after sulfonation (TPPTS) yielded much higher conversions than these with TPP (∼2.8–3.5 fold more).

**Table 1:** Homogeneous reactions in representative solvents^a^

Entry	Solvent	RP	Catalyst	Conversion (%)
1	Ethanol	0.654	PdCl_2_	81.4
2	Ethanol	0.654	Pd(OAc)_2_	54.4
3	Ethanol	0.654	PdCl_2_(TPP)_2_	15.7
4	Ethanol	0.654	Pd(OAc)_2_(TPP)_2_	19.7
5	Ethanol	0.654	PdCl_2_(TPPTS)_2_	44.6
6	Ethanol	0.654	Pd(OAc)_2_(TPPTS)_2_	55.0
7	Isopropanol	0.546	PdCl_2_(TPP)_2_	5.3
8	Isopropanol	0.546	PdCl_2_(TPPTS)_2_	7.0
9	Ethyl acetate	0.228	PdCl_2_(TPP)_2_	8.4
10	Ethyl acetate	0.228	PdCl_2_(TPPTS)_2_	14.2
11	Petroleum ether	0.117	PdCl_2_(TPP)_2_	5.2
12	Cyclohexane	0.006	PdCl_2_(TPP)_2_	4.2

a Reaction conditions: 0.5 mmol iodobenzene, 0.75 mmol phenylboronic acid, 10 μmol catalyst, 0.6 mmol Na_2_CO_3_, 5 mL solvent, 60 °C, 24 h.

As previously stated, the solvent has a profound effect on the catalytic performance in Suzuki cross-coupling reactions. Moreover, when heterogenized TMC is used as the catalyst of choice, and especially in the case where the complex is not chemically attached to the support, the solubility of the complex and the support in the solvent can lead to leaching and thus to a homogeneous reaction. Thereby, several representative organic solvents with different polarities, as illustrated by the relative polarity (RP) which was normalized from measurements of solvent shifts of absorption spectra,^50^ and relatively low boiling point that allow product separation and solvent recycling by distillation, were tested (Table 1). As expected, decreasing the polarity of the solvent, as illustrated by the decrease in RP, also decreased the conversion, probably due to lower solubility of phenylboronic acid and sodium carbonate in the solvents.

The effects of both the polysaccharide type and solvent type on the catalytic performance were also investigated (Table 2). Three commercial carrageenans were tested: ι, κ, λ-carrageenan, and a *Porphyridium* sp. (red microalga) soluble polysaccharide (P). This polysaccharide was chosen as it has different physicochemical structure, composition and sugar linkages, and different sulfate content than that of the carrageenan.^51^ The heterogeneous catalysts were added to the reaction mixture, using the more polar solvents, ethyl acetate, isopropanol and ethanol, and the reaction was performed under similar conditions as the homogeneous reaction. As can be seen in Table 2, the heterogeneous reaction conversions of the four polysaccharides with PdCl_2_(TPPTS)_2_ in ethanol, which is the most polar solvent, yielded the highest conversion rate in the Suzuki reaction, regardless of the polysaccharide type and structure.

**Table 2:** Heterogeneous reactions in representative solvents^a^

Entry	Solvent	Conversion (%)			
		κ	λ	ι	P
1	Ethyl acetate^b^	10.9	11.7	9.6	11.5
2	Isopropanol^b^	12.2	11.6	8.9	6.9
3	Ethanol^b^	77.8	55.2	71.9	63.3
4	Ethanol^c^	—	—	76.5	—
5	Ethanol^d^	—	—	80.0	—
6	Ethanol^e^	—	—	71.2	—
7	Ethanol^f^	—	—	99.0	—

a Reaction conditions: 0.5 mmol iodobenzene, 0.75 mmol phenylboronic acid, 10 μmol, 0.6 mmol Na_2_CO_3_, 5 mL solvent, 60 °C, 24 h.

b PdCl_2_(TPPTS)_2_.

c 10 μmol Pd(OAc)_2_(TPPTS)_2_.

d 10 μmol PdCl_2_(TPP)_2_.

e 10 μmol Pd(OAc)_2_(TPP)_2_.

f 10 μmol PdCl_2_.

The heterogeneous performances illustrated in Table 2 are in accordance with the conversions obtained in homogeneous reactions with the PdCl_2_(TPPTS)_2_ catalyst (Table 1), whereas the reactions in ethanol yielded the highest conversion rates. Notable is that the conversions of the heterogeneous systems with PdCl_2_(TPPTS)_2_ in ethanol were even higher than the conversions in the corresponding homogeneous reactions.^38^ This finding may imply that the carrageenan polysaccharides activated or stabilized the complex.^17,22^ It was suggested that the acceleration mechanism may be *via* stabilization of the complex by ligand–polysaccharide interactions, thus avoiding its deactivation due to the formation of palladium black by oxidation.^47^ Alternatively, the polar and protic characteristic of the polysaccharides, due to hydroxyl groups on the polymer backbone, may have produced a higher solubility rate of the phenylboronic acid and the base and/or a higher activity rate as was previously reported by the addition of water to a Suzuki cross-coupling reaction mixture of isopropanol^52^ or tetrahydrofuran (THF).^53^ Comparing the performance of the catalysts with results that were reported in literature, although reaction solvent and conditions as well as the substrates are different, shows that the activity rate is similar to that of other similar systems,^28,29,31^ but much lower than that of the systems that were tested under microwave irradiation, which is known to accelerate the reaction.^20,21,33–37^ Finally, the conversions that were observed when ethanol was used as a solvent in the heterogeneous reactions, were dependent on the polysaccharides type. These differences can be attributed to their different structures, sugar composition, sulfate groups content, and molecular weight, which probably yielded different matrix structures and different interactions with the complex.

The reaction conversion of various ι-based heterogenized palladium complexes in ethanol was also observed (Table 2, entries 3–6). Using ι as the polysaccharide and ethanol as the solvent, and changing the catalyst from PdCl_2_(TPPTS)_2_ to Pd(OAc)_2_(TPPTS)_2_ resulted in slightly higher conversion rates (Table 2, entries 3 and 4, respectively). Again, the conversion of both heterogeneous catalysts (Table 2, entries 3 and 4) was higher than that of the corresponding homogeneous reaction (Table 1, entries 5 and 6, respectively). Moreover, the heterogeneous catalyst Pd(OAc)_2_(TPPTS)_2_ yielded a higher conversion rate than the homogeneous Pd(OAc)_2_ without any ligand (Table 1, entry 2). Therefore, it can be concluded that the heterogeneous acceleration effect is not dependent on the type of the palladium salt (PdCl_2_/Pd(OAc)_2_) or the type of the ligand (TPP/TPPTS).

In order to check whether the reactions are heterogeneous or not and if the catalysts leached out from the support, the reaction mixtures were run for an additional 24 h under similar reaction conditions after removal of the catalyst by filtration. In all the heterogeneous reactions with PdCl_2_(TPPTS)_2_ (Table 2, entries 1–3) and Pd(OAc)_2_(TPPTS)_2_ (Table 2, entry 4), the conversions of the reaction after removal of the catalyst and mixing for an additional 24 h were not changed. Furthermore, ICP-MS detection of the reaction mixtures after 24 h reaction and removal of the heterogenous catalysts did not show any palladium leftovers, revealing that the reactions were indeed heterogeneous. However, using PdCl_2_, PdCl_2_(TPP)_2_ and Pd(OAc)_2_(TPP)_2_ (Table 2, entries 5–7, correspondingly) resulted in catalyst leaching, as manifested by the brown color of the reaction mixture and by the increase of conversion in the reaction mixture after removal of the catalyst support. These findings imply that using TPPTS as a ligand assists in the complex heterogenization. As stated before, FTIR analysis of i and ι-PdCl_2_(TPPTS)_2_ showed that the modification proceeded *via* the formation of a new bond between the TPPTS ligand with sulfonic acid sodium salt and the hydroxyl groups on the polysaccharide, yielding sulfonic esters, thus leading to heterogenization of the complex.^38^

Finally, the effect of substrate to catalyst (S/C) ratio on catalytic performance of the ι-PdCl_2_(TPPTS)_2_ catalyst was tested, changing both the amount of the substrate in the reaction mixture and the loading of the catalyst in the polysaccharide (Table 3). As can be seen in Table 3, increasing the substrate concentration and thus the S/C ratio linearly increased the TOF values, revealing that the catalyst can work in high capacity. Furthermore, changing the amount of the catalyst while keeping the amount of the substrate constant (entry 4), or increasing the amounts of the catalyst and the substrate while keeping the S/C ration constant (entry 5), also lead to the same trend. Finally, as expected, replacing iodobenzene with bromobenzene (entry 6) and chlorobenzene (entry 7) resulted in lower conversion rates, due to weaker reactivity of the halogen, as was also reported by others.^29,34^

**Table 3:** Effect of substrate (S) and catalyst (C) concentrations in Suzuki cross-coupling using ι-PdCl_2_(TPPTS)_2_ catalyst^a^

Entry	Halobenzene (mmol)	PdCl_2_(TPPTS)_2_ (μmol)	S/C	Conversion (%)	TOF (h^−1^)^b^
1	0.5^c^	10	50	71.9	1.5
2	1^c^	10	100	61.2	2.6
3	2^c^	10	200	56.3	4.7
4	0.5^c^	20	25	64.5	0.7
5	1^c^	20	50	60.3	1.3
6	0.5^d^	10	50	38.9	0.81
7	0.5^e^	10	50	21.1	0.44

a Reaction conditions: 50% excess phenylboronic acid, 20% excess Na_2_CO_3_, 5 mL ethanol, 60 °C, 24 h.

b TOF = (S/C) × conversion/24.

c Iodobenzene.

d Bromobenzene.

e Chlorobenzene.

Based on our previous report, PdCl_2_(TPPTS)_2_ supported on ι polysaccharide is the best candidate for catalyst recycling.^38^ Thus, the recycling of ι-PdCl_2_(TPPTS)_2_ catalyst was performed in ethyl acetate, isopropanol and ethanol, and the recycling of ι-Pd(OAc)_2_(TPPTS)_2_ catalyst was performed in ethanol (Table 4). As illustrated in Table 4, the catalysts can be recycled with only minor loss of activity. Moreover, changing the catalyst from ι-PdCl_2_(TPPTS)_2_ to ι-Pd(OAc)_2_(TPPTS)_2_ did not change the recycling efficiency.

**Table 4:** Catalyst recycling in representative solvents^a^

Entry	Conversion (%)			
Solvent	Ethyl acetate	Isopropanol	Ethanol	Ethanol
Catalyst	PdCl_2_(TPPTS)_2_	PdCl_2_(TPPTS)_2_	PdCl_2_(TPPTS)_2_	Pd(OAc)_2_(TPPTS)_2_
1	12.1	12.2	71.9	76.5
2	10.1	8.3	65.4	75.1
3	9.8	—	64.6	71.7
4	8.9	—	60.9	59.2
5	—	—	58.4	59.8

a Reaction conditions: 0.5 mmol iodobenzene, 0.75 mmol phenylboronic acid, 10 μmol catalyst, 0.6 mmol Na_2_CO_3_, 5 mL solvent, 50 °C, 24 h.

In order to understand whether the decrease in the catalyst activity is because of catalyst leaching, catalyst recycling analysis was performed after removal of the catalyst from the reaction mixture and addition of the catalyst to fresh reaction mixture with the corresponding amounts of fresh substrates and sodium carbonate. In addition, after removal of the catalyst, each reaction mixture was continued to run under similar conditions for an additional 24 h, to check whether the conversion increases with time, thus hinting whether the complex was leaching. Catalyst recycling analysis revealed that the conversion of the reaction did not increase after the removal of the catalyst and no palladium was observed in the solution by ICP-MS detection. Thus, the loss of conversion can be attributed to deactivation of the complex or to loss of some catalyst during the separation step.

**Fig. 2:**
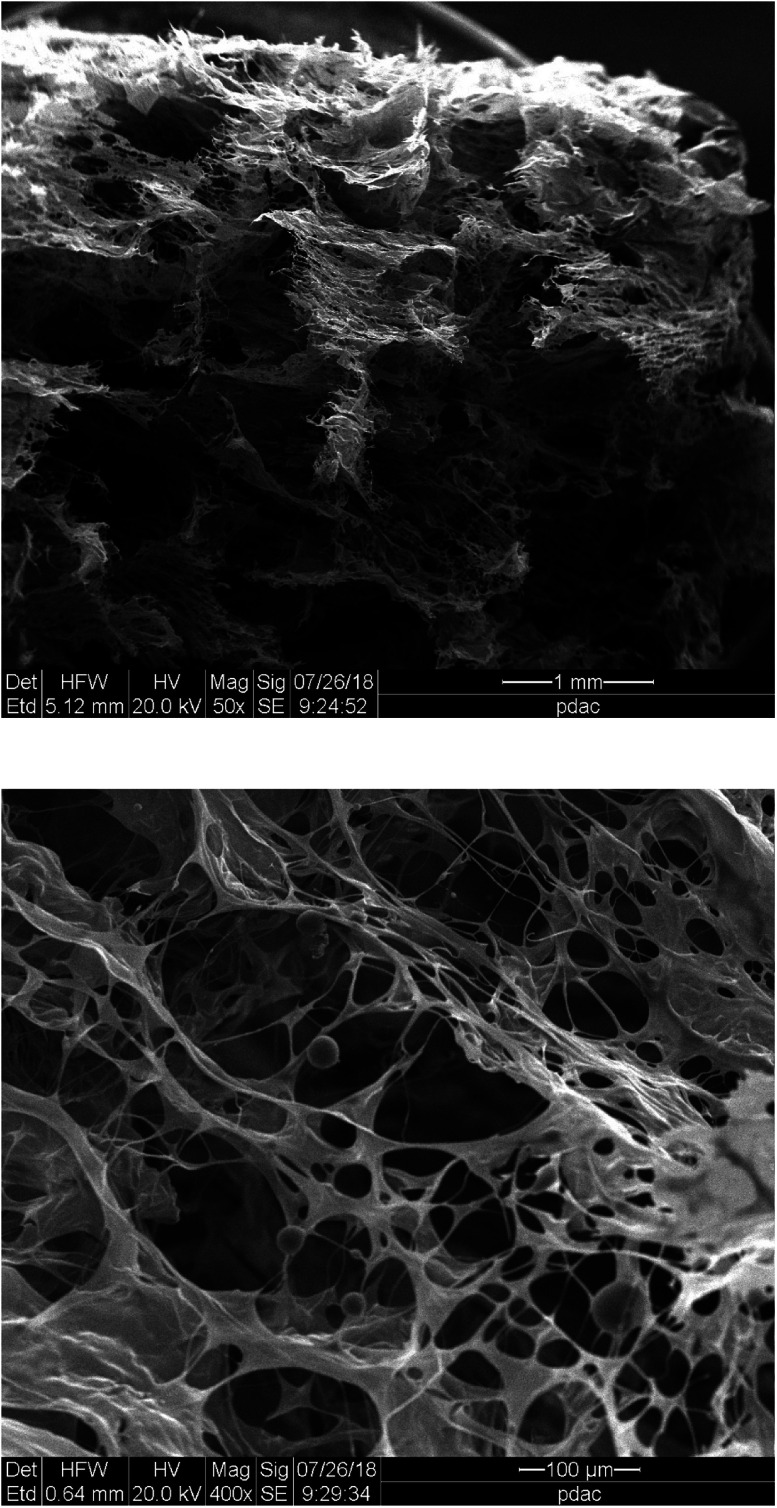
SEM images of ι-Pd(OAc)_2_(TPPTS)_2_.

The final stage of the study was to gain more knowledge regarding the structural characterization of the ι-Pd(OAc)_2_(TPPTS)_2_ heterogeneous catalyst. Thus, the heterogeneous system was first analysed using SEM, and the SEM image of the lyophilized ι-Pd(OAc)_2_(TPPTS)_2_ system showed that it is characterized by porous sphere-hollows structure with different sizes (Fig. 2). Moreover, comparing the structures of ι-Pd(OAc)_2_(TPPTS)_2_ with previous reported results of ι-PdCl_2_(TPPTS)_2_ system that has reticular porous arrangement^38^ show that changing the palladium salt yield different pore size and structure. Thus, it was suggested that the different Pd salts, *i.e.* acetate or chloride, interacted differently with the polysaccharide matrix, probably in accordance to their different size and charge distribution.

**Fig. 3:**
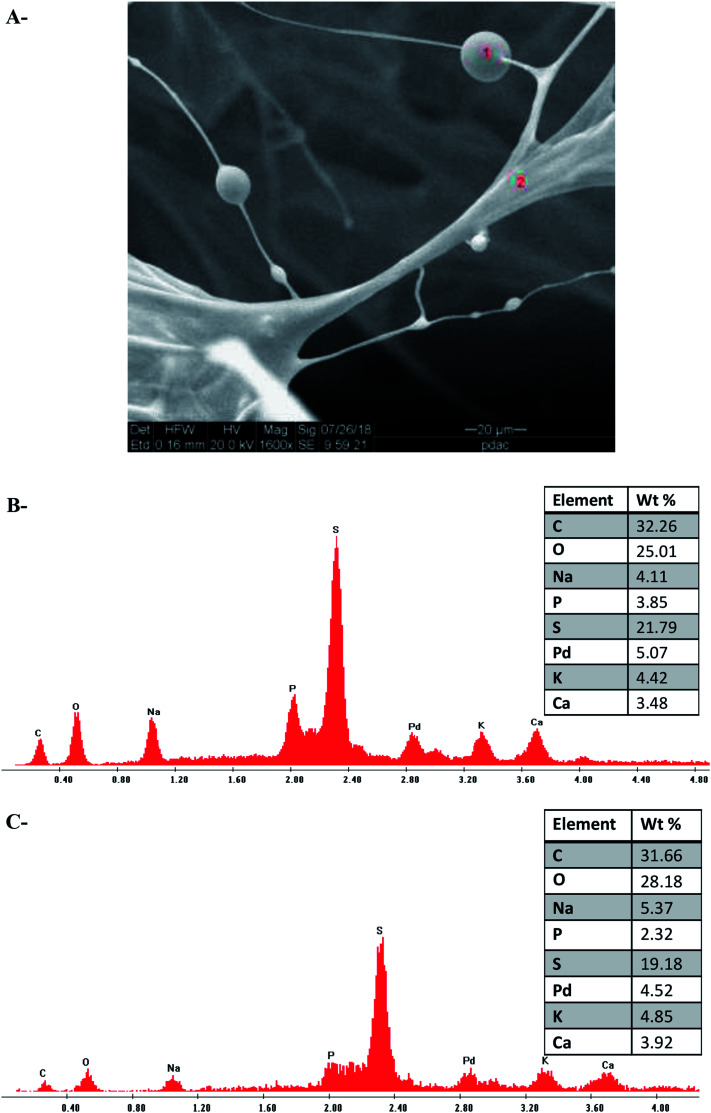
SEM-EDS analysis of Pd(OAc)_2_(TPPTS)_2_ supported on ι (A) SEM image (B and C) EDS spectrum and tabulated results of the sections that are signed in red numbers.

In the next stage, an elemental analysis of ι-Pd(OAc)_2_(TPPTS)_2_ was performed, using SEM-EDS. Representative EDS spectrum of the sample revealed that all the expected elements, which were part of the heterogeneous catalyst system are also observed in the final sponge-like structure (Fig. 3A–C). In addition, as expected, the molar elemental ratio of P : Pd in the heterogeneous catalyst was around 2 : 1, as in the homogenous complex, as illustrated in Fig. 3B and C, respectively.

**Fig. 4:**
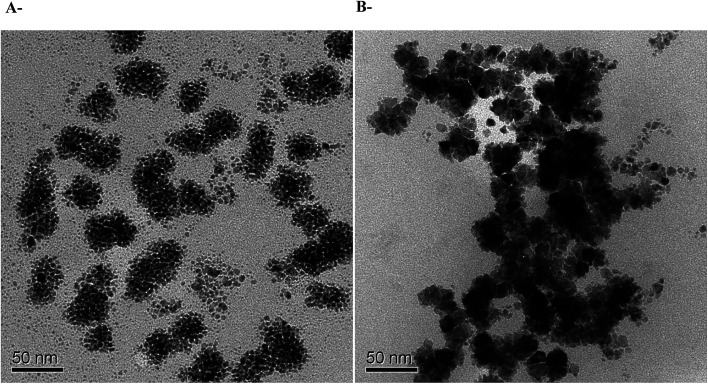
TEM micrographs of (A) fresh ι-Pd(OAc)_2_ (TPPTS)_2_; (B) ι-Pd(OAc)_2_ (TPPTS)_2_ after reaction.

Owing to their very safe and stable biodegradation and biocompatibility, polysaccharides are considered as very promising hosts for metallic nanoparticles.^54–56^ In addition, they are especially attractive due to their high surface area to volume ratios and high surface energy, which allow accessibility to the metal sites.^57^ Therefore, in order to study whether ι-Pd(OAc)_2_(TPPTS)_2_ system is composed of nanoparticles as observed in our former study for ι-Pd(Cl)_2_(TPPTS)_2_ system,^38^ TEM analysis of the fresh catalyst and the catalyst after reaction were also performed (Fig. 4). The TEM image of the heterogeneous ι-Pd(OAc)_2_(TPPTS)_2_ catalyst before the reaction (Fig. 4A), shows that nanoparticles were created already during the lyophilization of the water soluble complex and the polysaccharide solution. However, employing the catalysts in the reaction and heating it for 24 h at 60 °C, resulted in the formation of much bigger nanoparticles, as illustrated in Fig. 4B. It implies that the reaction conditions promote the aggregation of nanoparticles as well as that the complex was reduced under reaction conditions.^38^ Moreover, these nanoparticles might be the reason for the slight reduction in the catalyst activity during recycling cycles (Table 4). In addition, these findings are in accordance to those observed with ι-PdCl_2_(TPPTS)_2_ system that was made by the same procedure.

**Fig. 5:**
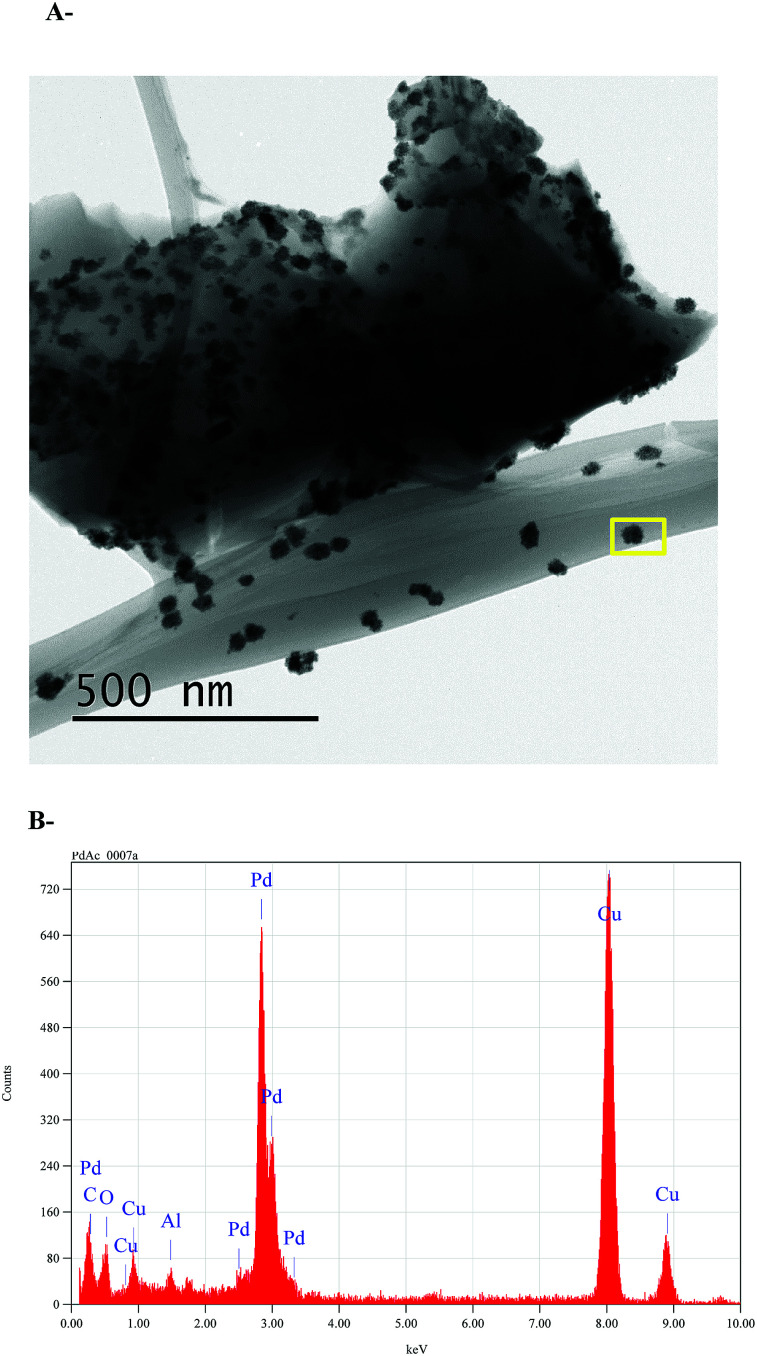
Pd nanoparticles on ι-Pd(OAc)_2_ (A) TEM micrographs, (B) TEM-EDS analysis. The yellow rectangle in the inset image in Fig. 5A shows the selected EDS inspection field.

Finally, in order to investigate whether the nanoparticles are produced due to the TPPTS ligand content, a TEM analysis of ι-Pd(OAc)_2_ system that was prepared in the same procedure was also performed (Fig. 5). It was found that nanoparticles with similar sizes and shape are also formed, in regardless to the TPPTS ligand. Yet, it is important to note that using ι-Pd(OAc)_2_ as catalysts in Suzuki cross-coupling in ethanol resulted in leaching of the palladium to the reaction mixture.^38^ It reveals that the ligand is necessary to ensure heterogenization of the complex on the polysaccharide, and that the interaction of the complex to the support is *via* the ligand. In addition, elemental analysis using TEM-EDS of the nanoparticles demonstrated that they are based of Pd (Fig. 5B). Indeed, several studies have proven that palladium nanoparticles can be prepared using porous polysaccharides as supports, *i.e.* palladium-supported on derived-polysaccharides such as starch,^22^ glucomannans^58^ and exopolysaccharides produced by bacteria cells.^59^

## Conclusions

To conclude, red algae-derived polysaccharides, composed of hydroxyl and sulfate groups, were successfully used to heterogenize palladium based complexes in the Suzuki cross-coupling of halobenzenes and phenylboronic acid. The heterogenization procedure described herein is very simple and straightforward, and can be employed to immobilize various TMCs for different reactions. In addition, the procedure is very versatile and the type of the polysaccharide, the TMC and the solvent can be tailored for each system.

The reactions in ethanol using PdCl_2_(TPPTS)_2_ and Pd(OAc)_2_(TPPTS)_2_ with ι as the polysaccharide showed high activity rates due to the polarity of the solvent. Moreover, the complex did not leach out to the reaction mixture and the catalyst was successfully recycled with minor loss of activity. Among all the solvents that were tested, ethanol is very attractive due to its environmentally friendly character and relatively low boiling point, which allows the separation of the product by distillation. In addition, not only did the combination of polysaccharides with palladium complexes result in heterogeneous recyclable catalysts, it also activated the complexes, thus yielding conversion rates that were higher than those of the homogeneous systems. Furthermore, the characterization of the lyophilized ι-Pd(OAc)_2_(TPPTS)_2_ system showed that it is characterized by a porous sphere-hollow structure with different sizes. Additionally, TEM analysis of the fresh catalyst showed that nanoparticles were created already during the lyophilization of the water soluble complex and the polysaccharide solution, while under reaction conditions much larger nanoparticles were formed. This implies that the reaction conditions promote the aggregation of nanoparticles and that the complex was reduced under reaction conditions. At last, it was also proven that both Pd(OAc)_2_(TPPTS)_2_ and Pd(OAc)_2_ supported on ι in the technique that was reported herein produced nanoparticles, yet only the complex with the ligand prevented leaching. Nonetheless, the metal nanoparticles formation in these systems can serve in various fields including catalysis, optics, images, diagnosis and nanomedicine.

## Conflicts of interest

There are no conflicts to declare.

## Supplementary Material
